# Evaluation of the Biological Properties and Antibacterial Activities of the Natural Food Supplement “Epavin” for Liver Detoxification and Protection

**DOI:** 10.3390/foods14152600

**Published:** 2025-07-24

**Authors:** Alexia Barbarossa, Maria Pia Argentieri, Maria Valeria Diella, Anita Caforio, Antonio Carrieri, Filomena Corbo, Antonio Rosato, Alessia Carocci

**Affiliations:** Department of Pharmacy-Pharmaceutical Sciences, University of Bari “Aldo Moro”, Via E. Orabona, 4, 70125 Bari, Italy; mariapia.argentieri@uniba.it (M.P.A.); m.diella2@phd.uniba.it (M.V.D.); anita.caforio29@gmail.com (A.C.); antonio.carrieri@uniba.it (A.C.); filomena.corbo@uniba.it (F.C.); antonio.rosato@uniba.it (A.R.)

**Keywords:** hepatotoxicity, antioxidants, heavy metals, antibacterial activity, bioactive compounds

## Abstract

Background/Objectives: The liver, the body’s primary detoxifying organ, is often affected by various inflammatory diseases, including hepatitis, cirrhosis, and non-alcoholic fatty liver disease (NAFLD), many of which can be exacerbated by secondary infections such as spontaneous bacterial peritonitis, bacteremia, and sepsis—particularly in patients with advanced liver dysfunction. The global rise in these conditions underscores the need for effective interventions. Natural products have attracted attention for their potential to support liver health, particularly through synergistic combinations of plant extracts. *Epavin*, a dietary supplement from Erbenobili S.r.l., formulated with plant extracts like *Taraxacum officinale* (L.), *Silybum marianum* (L.) *Gaertn.*, and *Cynara scolymus* (L.), known for their liver-supporting properties, has been proposed as adjuvant for liver functions. The aim of this work was to evaluate of *Epavin*’s antioxidant, anti-inflammatory, and protective effects against heavy metal-induced toxicity. In addition, the antibacterial effect of *Epavin* against a panel of bacterial strains responsible for infections associated with liver injuries has been evaluated. Methods: The protection against oxidative stress induced by H_2_O_2_ was evaluated in HepG2 and BALB/3T3 cells using the dichlorofluorescein diacetate (DCFH-DA) assay. Its anti-inflammatory activity was investigated by measuring the reduction in nitric oxide (NO) production in LPS-stimulated RAW 264.7 macrophages using the Griess assay. Additionally, the cytoprotecting of *Epavin* against heavy metal-induced toxicity and oxidative stress were evaluated in HepG2 cells using the [3-(4,5-dimethylthiazol-2-yl)-2,5-diphenyl-tetrazolium bromide] (MTT) and DCFH-DA assays. The antibacterial activity of *Epavin* was assessed by determining the minimum inhibitory concentration (MIC) and the minimum bactericidal concentration (MBC) against Gram-positive (*Enterococcus faecalis* ATCC 29212, and BS, *Staphylococcus aureus* 25923, 29213, 43300, and BS) and Gram-negative (*Escherichia coli* 25922, and BS, *Klebsiella pneumoniae* 13883, 70063, and BS) bacterial strains using the microdilution method in broth, following the Clinical and Laboratory Standards Institute’s (CLSI) guidelines. Results: *Epavin* effectively reduced oxidative stress in HepG2 and BALB/3T3 cells and decreased NO production in LPS-stimulated RAW 264.7 macrophages. Moreover, *Epavin* demonstrated a protective effect against heavy metal-induced toxicity and oxidative damage in HepG2 cells. Finally, it exhibited significant antibacterial activity against both Gram-positive and Gram-negative bacterial strains, with MIC values ranging from 1.5 to 6.0 mg/mL. Conclusions: The interesting results obtained suggest that *Epavin* may serve as a valuable natural adjuvant for liver health by enhancing detoxification processes, reducing inflammation, and exerting antibacterial effects that could be beneficial in the context of liver-associated infections.

## 1. Introduction

The liver plays a crucial role in maintaining metabolic homeostasis, detoxifying xenobiotics, and regulating immune responses; thus, its proper function is essential for overall health [1]. However, chronic liver diseases (CLDs), including inflammatory hepatic conditions, such as non-alcoholic fatty liver disease (NAFLD) and alcoholic liver disease (ALD), represent a significant global health burden, contributing to substantial morbidity and mortality worldwide. Oxidative stress is a key player in the pathogenesis of CLDs and can promote the development of serious complications, including hepatocarcinogenesis [2]. Furthermore, the systemic inflammation associated with decompensated cirrhosis and liver failure can impair immune function, increasing patients’ susceptibility to bacterial infections and further deteriorating their condition [3]. These infections are frequently sustained by specific bacterial pathogens, including *Enterococcus faecalis*, *Staphylococcus aureus*, *Escherichia coli* (including ESBL-producing strains), and *Klebsiella pneumoniae.* Such microorganisms are commonly implicated in spontaneous bacterial peritonitis (SBP), bacteremia, and urinary tract infections in patients with end-stage liver disease or cirrhosis [4]. Despite significant progress in modern medicine, there are still no fully effective drugs that can enhance liver function, provide comprehensive protection to the organ, or promote hepatic cell regeneration [5]. Moreover, some drugs can induce adverse or side effects [6]. In recent years, increasing attention has also been given to the hepatotoxic effects of environmental pollutants, particularly heavy metals such as lead (Pb), cadmium (Cd), and mercury (Hg). These toxic elements, commonly encountered through contaminated water, food, or industrial exposure, can accumulate in hepatic tissues and disrupt critical cellular functions. Heavy metals are known to induce oxidative stress, mitochondrial dysfunction, and inflammatory responses, ultimately contributing to hepatocyte damage, fibrosis, and impaired detoxification capacity [7]. Their presence can also exacerbate pre-existing liver conditions and complicate clinical outcomes [8]. Thus, there is a growing interest in identifying supportive strategies that can aid liver protection and the restoration of hepatic function [9,10]. Among the promising approaches, natural compounds and botanical extracts have attracted attention for their potential hepatoprotective, antioxidant, anti-inflammatory, and antibacterial properties [11,12]. These natural products often exhibit a multi-targeted mode of action and, when used in combination, may act synergistically to enhance their efficacy [13]. In particular, multi-herbal formulations are being investigated for their ability to modulate key molecular pathways involved in liver injury and repair [14]. One of the most common and accessible means of administering these bioactive plant compounds is through nutraceuticals or dietary supplements. The use of such formulations is increasingly widespread among individuals seeking preventive or complementary approaches to support liver health [15]. These supplements often contain standardized extracts, ensuring consistent concentrations of active constituents, and are generally well tolerated [16]. Their oral administration allows for ease of use in daily routines, providing a practical strategy for delivering biologically active compounds that may support detoxification processes, reduce oxidative damage, and promote overall liver function [17,18]. In addition to their hepatoprotective potential, the antimicrobial properties of botanical extracts may be particularly relevant in the context of chronic liver diseases. Individuals with advanced hepatic dysfunction often develop immunological alterations that compromise their ability to contain bacterial translocation and systemic infections. For example, cirrhotic patients exhibit increased intestinal permeability and impaired immune surveillance, factors that contribute to the development of spontaneous bacterial peritonitis, bacteremia, and urinary tract infections [19]. Therefore, formulations that combine antioxidant, hepatoprotective, and antibacterial activities may offer a comprehensive approach to mitigate both hepatic injury and infection-related complications [20]. However, despite growing interest in polyherbal formulations, the scientific evidence supporting the efficacy of such combinations—especially in comparison to their individual constituents—remains limited. Few studies have systematically assessed the biological activity of commercial plant-based supplements in hepatic models, and even fewer have evaluated whether the combined effects exceed or differ from those of their key bioactive compounds [21]. The dietary supplement object of this study is *Epavin*. Its formulation includes a combination of medicinal plant extracts traditionally used for hepatic detoxification and protection. Among them, *Silybum marianum* (milk thistle) is widely recognized for its hepatoprotective properties, primarily due to the presence of silymarin, a mixture of flavonolignans extracted from plant seeds [22]. Silymarin is composed mainly of silybin A and B (collectively referred to as silibinin), isosilybin A and B, silychristin, and silydianin. Among these, silybin represents the most abundant and pharmacologically active constituent. These flavonolignans have been shown to exert antioxidant, anti-inflammatory, and membrane-stabilizing effects, protecting hepatocytes from toxins and supporting cellular regeneration [23]. *Cynara scolymus* L. (artichoke) extract promotes bile flow (choleresis), aids digestion, and demonstrates hepatoprotective and lipid-lowering properties, potentially through modulation of oxidative stress and inflammatory mediators [24]. *Taraxacum officinale* (L.) (dandelion) has traditionally been used for its choleretic, diuretic, and mild anti-inflammatory effects, contributing to the facilitation of liver detoxification and overall hepatic function [25]. The aim of our study was to investigate the effects of the combination of these compounds, in the form of *Epavin*, on hepatocytes function and protection. In particular, we explored in vitro the hepatoprotective role of *Epavin* against pro-inflammatory and pro-oxidative stimuli as well as its role in counteracting bacterial infections. Additionally, we compared the effects of Epavin to those of its principal phenolic component, chlorogenic acid (CGA), in order to evaluate whether the formulation provides enhanced biological activity relative to its main constituent.

## 2. Materials and Methods

### 2.1. Epavin

The food supplement Epavin was supplied by Erbenobili s.r.l. (Corato, Italy). It is a hydroalcoholic formulation composed of standardized herbal extracts traditionally used for liver support. According to the manufacturer’s declaration, the formulation includes 20% (plant/solvent ratio) hydroalcoholic extracts (water and ethanol) of the following botanicals: dandelion (*Taraxacum officinale* (L.) Weber ex F.H.Wigg.) root, milk thistle (*Silybum marianum Gaertn.*) seeds, artichoke (*Cynara scolymus* L.) leaves, barberry (*Berberis vulgaris* L.) bark of the branches, boldo (*Peumus boldus Molina*) leaves, black radish (*Raphanus sativus* L.) roots, Chinese rhubarb (*Rheum palmatum* L.) root, American crisantello (*Chrysantellum americanum* (L.) Vatke) whole plant, desmodium (*Desmodium adscendes* (SW.)DC.) leaves, and combreto (*Combretum G. micranthum Fr.*) leaves. The essential oils are derived from peppermint (*Mentha piperita* L.) leaves and rosemary (*Rosmarinus officinalis* L.) leafy branch.

### 2.2. Chemicals

The following chemicals were purchased from the Sigma-Aldrich S.p.a. (Milan, Italy): [3-(4,5-dimethylthiazol-2-yl)-2,5-diphenyl-tetrazolium bromide] (MTT), nicotinamide adenine dinucleotide phosphate (NADPH), ethylenediaminetetraacetic acid sodium salt (Na-EDTA), sodium pyruvate, hydrogen peroxide, CdCl_2_, HgCl_2_, Pb(NO_3_)_2_, L-glutamine, trypsin, and 2′,7′-dichlorofluorescein diacetate. The following cell culture materials were purchased: high-glucose (4.5 g L^−1^) Dulbecco’s modified Eagle medium (DMEM) and fetal bovine serum (FBS, Euroclone S.p.A., Pero, MI, Italy). All other chemicals were of the highest analytical grade and purchased from common sources.

### 2.3. Chemical Characterization

Phenolic acids and flavonoids from *Epavin* were quantitatively analyzed using a Waters 600 HPLC system equipped with a photodiode array detector (PDA) and a Gemini C18 column (Phenomenex, Torrance, CA, USA; 250 × 4.6 mm, 5 μm particle size). The mobile phase consisted of solvent A (water with 0.1% formic acid) and solvent B (acetonitrile with 0.1% formic acid). The elution gradient initiated at 10% B, increased linearly to 40% at 40 min, and reached 60% B at 60 min. The flow rate was maintained at 1 mL/min, and the injection volume was 20 μL. UV spectra were recorded at 210, 280, 310, and 350 nm. All analyses were conducted in triplicate [26].

Quantification of phenolic acids was performed using a calibration curve generated from chlorogenic acid standards (Sigma-Aldrich) across eight concentrations within a linear range of 31.25–1000 μg/mL. The calibration curve showed a strong linear relationship (R^2^ = 0.9992) with the equation y = 5 × 10^7^x − 696495.

Flavonoid content was determined using a calibration curve based on cinaroside (Extrasynthese), prepared at five concentrations ranging from 31.25 to 500 μg/mL. The resulting curve had a correlation coefficient of R^2^ = 0.9997 and the equation y = 3 × 10^7^x − 335339.

Compound identification was carried out using an Agilent 1100 Series LC/MSD Trap System VL, with data acquisition and processing performed using Agilent Chemstation software (LC/MSD Trap Software 4.1; Agilent Technologies, Santa Clara, CA, USA, 2002). Analyses were conducted using an electrospray ionization (ESI) source operated in both positive and negative ion modes under the following conditions: capillary voltage set at 4000 V, nebulizer gas (nitrogen) pressure at 15 psi, drying gas (nitrogen) temperature at 350 °C, and flow rate at 5 L/min.

Full scan mass spectra were acquired over an *m*/*z* range of 100–2200, at a scan rate of 13,000 *m*/*z* per second. Automated ESI-MS/MS was performed by isolating molecular ions (base peaks) with an isolation width of 4.0 *m*/*z*, a minimum intensity threshold of 100, and ion charge control enabled, with a maximum acquisition time of 300 ms. MS/MS fragmentation was achieved using variable collision energies of 1.0, 10.0 and 30.0 V.

The extracts were dissolved in methanol (MeOH) at concentrations ranging from 20 to 30 ppm and introduced into the system at a flow rate of 10 μL/min using a KD Scientific Syringe Pump (KD Scientific Inc., Holliston, MA, USA).

### 2.4. Cell Culture

Human hepatocellular liver carcinoma (HepG2) cells were purchased from the American Type Culture Collection (ATCC, Manassas, VA, USA) and maintained at 37 °C in a humidified atmosphere (95% air and 5% carbon dioxide). The cells were cultured in Eagle’s Minimum Essential Medium (MEM, Euroclone S.p.A., Pero, MI, Italy), supplemented with 10% Fetal Bovine Serum (FBS, Euroclone S.p.A., Pero, MI, Italy), 1% L-glutamine (Euroclone S.p.A., Pero, MI, Italy), 100 U/mL penicillin/streptomycin (Euroclone S.p.A., Pero, MI, Italy), and 1% Non-Essential Amino Acids (NEAA, Euroclone S.p.A., Pero, MI, Italy). The cells were periodically screened for mycoplasma contamination by means of MycoSEQ Mycoplasma Detection Assay (Thermo Fisher Scientific, Waltham, MA, USA).

Murine fibroblast BALB/c 3T3 clone A31 cells were also obtained from ATCC and cultured under similar conditions. The cells were maintained in Dulbecco’s Modified Eagle Medium (DMEM, Euroclone S.p.A., Pero, MI, Italy), supplemented with 10% FBS, 1% L-glutamine, and 100 U/mL penicillin/streptomycin.

Murine macrophage RAW 264.7 cells were acquired from ATCC and maintained in DMEM, supplemented with 10% FBS, 1% L-glutamine, and 100 U/mL penicillin/streptomycin. The cells were cultured at 37 °C in a humidified atmosphere containing 5% CO_2_ and 95% air. The medium was refreshed every 2 days, and the cells were subcultured as needed based on confluency and experimental timing [27].

### 2.5. Evaluation of Cytotoxicity Using MTT Assay

The MTT (3-(4,5-dimethylthiazol-2-yl)-2,5-diphenyltetrazolium bromide) assay was used to assess cell viability. HepG2 cells were seeded into 96-well plates at a density of 5 × 10^3^ cells/well and allowed to adhere overnight. To evaluate the potential cytotoxicity of *Epavin* and chlorogenic acid (CGA) alone, the cells were treated with increasing concentrations of *Epavin* (125, 250 and 500 µg/mL) or CGA (25, 50 and 100 µg/mL) for 24 h. After treatment, 20 µL of MTT solution (5 mg/mL in PBS) was added to each well, and the plates were incubated at 37 °C for 3 h. Formazan crystals were solubilized by adding 100 µL of DMSO, and absorbance was measured at 570 nm using a microplate reader Tecan Infinite M1000 Pro (Tecan, Cernusco S.N., Italy) [28].

To evaluate the cytotoxicity of heavy metals, the cells were treated for 24 h with 30 µM cadmium chloride (CdCl_2_), mercuric chloride (HgCl_2_), or lead nitrate (Pb(NO_3_)_2_). Additionally, to assess the cytoprotective effects of *Epavin* or CGA, the cells were co-treated with the above-mentioned concentrations of *Epavin* or CGA, followed by 24 h co-treatment with each heavy metal at 30 µM. After treatment, the MTT assay was carried out as described above.

All treatments were performed in triplicate and repeated in at least three independent experiments. Cell viability was expressed as a percentage relative to untreated control cells.

### 2.6. Measurement of Intracellular ROS Production

Intracellular reactive oxygen species (ROS) generation was quantified using the 2′,7′-dichlorofluorescein diacetate (DCFH-DA) assay in HepG2 and BALB 3T3 cells subjected to oxidative stress following a slightly modified protocol from the literature [29]. For H_2_O_2_-induced ROS production, the cells were seeded in black 96-well plates at a density of 5 × 10^3^ cells/well and incubated overnight. After cell attachment, treatments were performed with *Epavin* (125, 250, 500 µg/mL) or chlorogenic acid (CGA; 25, 50, 100 µg/mL) for 1 h, followed by exposure to 50 µM H_2_O_2_ for 30 min. DCFH-DA (final concentration 25 µM) was added during the last 30 min of incubation. The cells were then washed with PBS to remove excess dye, and fluorescence was measured at 485 nm excitation and 535 nm emission using a microplate reader Tecan Infinite M1000 Pro (Tecan, Cernusco S.N., Italy). For ROS measurements under heavy metal-induced stress, only HepG2 cells were used. After seeding and overnight incubation, the cells were pretreated with *Epavin* or CGA at the same concentrations as above for 1 h, followed by co-treatment with 30 µM of cadmium chloride (CdCl_2_), mercuric chloride (HgCl_2_), or lead nitrate (Pb(NO_3_)_2_) for 12 h. DCFH-DA (10 µM) was added during the final 30 min of incubation. Following treatment, the cells were washed with PBS and fluorescence intensity was recorded as described above. All conditions were tested in triplicate in three independent experiments. Fluorescence values were normalized to untreated controls and expressed as percentage of ROS production relative to stress-induced groups.

### 2.7. Evaluation of Anti-Inflammatory Activity Using the Griess Assay

To assess the anti-inflammatory potential of *Epavin* and its main phytochemical constituent, chlorogenic acid (CGA), nitric oxide (NO) production was measured in LPS-stimulated RAW 264.7 macrophages using the Griess assay. RAW 264.7 cells were seeded in 96-well plates at a density of 1 × 10^5^ cells/well and incubated at 37 °C in a humidified atmosphere with 5% CO_2_ for 24 h. After attachment, the cells were pre-treated with various concentrations of *Epavin* (125, 250 and 500 µg/mL) or CGA (25, 50 and 100 µg/mL) for 1 h. Lipopolysaccharide (LPS) was then added to each well at a final concentration of 1 µg/mL to induce NO production, and the cells were incubated for an additional 24 h. At the end of the incubation period, 100 µL of the culture supernatant was collected from each well and mixed with an equal volume of Griess reagent (Sigma-Aldrich, St. Louis, MO, USA) according to the manufacturer instructions. The mixture was incubated at room temperature for 10 min, and absorbance was measured at 540 nm using a microplate reader Tecan Infinite M1000 Pro (Tecan, Cernusco S.N., Italy). Nitrite concentration, used as an indicator of NO production, was calculated based on a standard curve of sodium nitrite. All experiments were performed in triplicate and repeated independently at least three times.

### 2.8. Antibacterial Studies

The antibacterial activity of *Epavin*, chlorogenic acid (CGA), and the reference antibiotic levofloxacin was assessed following the broth microdilution method described in the Clinical and Laboratory Standards Institute (CLSI) guidelines (M07-A9, 2012) [12]. The assay included both reference and clinical bacterial strains. Gram-positive strains consisted of *Staphylococcus aureus* ATCC 25923, ATCC 29213, ATCC 43300 (methicillin-resistant), and a clinical isolate (*S. aureus* BS, *acronym for Civil hospital* “*Casa della Divina Provvidenza*”, *Bisceglie*, *Bari*, *Italy*), as well as *Enterococcus faecalis* ATCC 29212 and a clinical isolate (*E. faecalis* BS). Gram-negative strains included *Escherichia coli* ATCC 25922, a clinical ESBL-producing *E. coli* isolate (*E. coli* BS), *Klebsiella pneumoniae* ATCC 13883, ATCC 700603, and a clinical isolate (*K. pneumoniae* BS). Clinical strains were isolated from positive blood cultures of hospitalized patients and were kindly provided by the Hygiene Section, Department of Biomedical Sciences and Human Oncology, University of Bari, Italy, and identified by standard physiological and morphological methods (API systems: API 20S, API RapidStaph, API Rapid 20E). In general, the clinical isolates of *E. coli* and *K. pneumoniae* displayed resistance to multiple antibiotic classes, including penicillins and aminoglycosides, consistent with their ESBL-producing phenotype. Reference strains from the American Type Culture Collection (ATCC) were used as quality controls, with susceptibility profiles established by CLSI standards. CGA and levofloxacin were initially dissolved in DMSO at the highest possible concentration (20 mg/mL) and subsequently diluted in Cation-Adjusted Mueller–Hinton Broth (CAMHB) to achieve the desired test concentrations, whereas *Epavin* was directly dissolved and serially diluted in CAMHB. Bacterial suspensions were prepared by incubating each strain in Mueller–Hinton Broth (MHB) at 37 °C for 3–5 h. The turbidity was adjusted to a 0.5 McFarland standard (OD625 = 0.08–0.10), and the suspension was then diluted 1:100 to achieve a final inoculum of ~1–2 × 10^6^ CFU/mL. An aliquot of 200 µL was added to each well of the microplate. The plates were incubated at 37 °C for 24 h. MIC was defined as the lowest concentration at which no visible bacterial growth was observed. For MBC determination, 10 µL from each clear well was plated on Mueller–Hinton agar and incubated for an additional 24 h. MBC was determined as the lowest concentration that resulted in no visible bacterial colonies, indicating a ≥99.9% reduction in viable bacteria. Levofloxacin was used as the reference antibiotic. All experiments were performed three times in duplicate to ensure reproducibility.

### 2.9. Statistical Analysis

All the data are presented as mean ± standard deviation (SD), with values derived from a minimum of three independent experiments. All the assays were performed in triplicate to ensure reproducibility. Statistical significance (*p* < 0.0001) was assessed using a one-way ANOVA followed by Dunnett’s post hoc test in GraphPad Prism 9.0. Statistical significance was defined as a *p*-value ≤ 0.05.

## 3. Results

### 3.1. Epavin Chemical Composition

**Figure 1:**
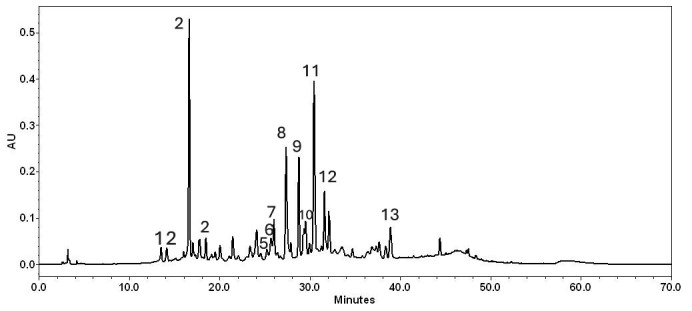
HPLC-DAD chromatogram of Epavin at 350 nm. The number of peaks aligns with that listed in Table 1.

The chemical composition of *Epavin* was checked by high performance liquid Chromatography (HPLC)-diode array detection (DAD) and HPLC high-resolution mass spectrometry (HRMS). Table 1 summarizes the UV and mass spectral data for each compound identified. Several components were identified through direct comparison with commercial reference standards, while other phenolic compounds were characterized based on interpretation of their UV and mass spectra, supported by previously published literature data [30]. A combination of HPLC-PDA and LC-MS analyses (Table 1) confirmed the presence of both flavonoids and phenolic acids in *Epavin*. UV spectral profiles of the eluted compounds revealed two major absorption bands: one in the 240–280 nm range, corresponding to the A-ring (benzoyl system, Band II), and another in the 330–350 nm range, associated with the B-ring (cinnamoyl system, Band I). These absorption features are characteristic of flavone structures. Additionally, compounds displaying absorption maxima between 325.7 and 329.3 nm and 246.1–250.0 nm, along with a diagnostic sharp shoulder at 290–300 nm, were definitively identified as chlorogenic acids derivatives. Moreover, the UV maxima around 270–290 nm and 300–330 nm suggested the presence of flavolignans derivatives. Quantitation indicated that the main constituents (Figure 1) identified in *Epavin* were phenolic acids, representing approximately 55%; in particular, chlorogenic acid and 3,4-dicaffeoylquinic acid were the most abundant. In the food supplement, flavonoids had also been identified (33%), and among these, cynaroside was the most abundant. As expected, in *Epavin*, flavolignans such as silicristin, silydianin, and silybin A and B were identified. Silybin A was more abundant than the others. The mixture of these compounds, known as silymarin, represents about 12%.

### 3.2. ROS Scavenging Effects of Epavin Against H_2_O_2_-Induced Oxidative Stress

**Figure 2:**
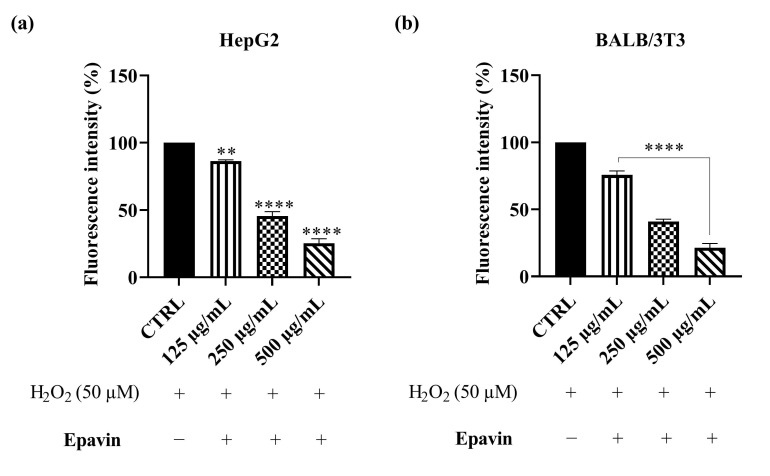
DCFH oxidation in HepG2 (**a**) and BALB-3T3 (**b**) cells after exposure to H_2_O_2_, 50 μM, and different concentrations of *Epavin* (125–500 µg/mL). “+” indicates the presence and “−” the absence of treatment. The results are shown as mean ± standard deviation (SD) (*n* = 3). Significant differences versus CTRL: ** *p* < 0.01, and **** *p* < 0.0001.

To evaluate intracellular ROS levels in the absence or presence of *Epavin* at different concentrations (125, 250 and 500 μg/mL), the DCFH-DA assay was performed on HepG2 and BALB 3T3 cells after stimulation with 50 μM H_2_O_2_. These two cell models were chosen due to their different biological characteristics: HepG2 cells, derived from human hepatocellular carcinoma, represent a model for studying oxidative stress and antioxidant responses in liver cells, while BALB 3T3, a murine fibroblast cell line, serves as a model for evaluating general cytoprotective effects and oxidative stress responses in non-tumorigenic cells. A preliminary MTT assay was performed in order to rule out the potential cytotoxicity of *Epavin* in both cell lines used in our studies. The results confirmed that *Epavin* did not significantly affect cell viability up to the highest tested concentration of 500 μg/mL, thereby validating the use of these concentrations in subsequent experiments. As shown in Figure 2, the results obtained with *Epavin* demonstrate a dose-dependent antioxidant effect in both cell lines, suggesting its potential role in mitigating oxidative stress in different cellular contexts. In particular, in HepG2 cells, treatment with 125 μg/mL of *Epavin* reduced ROS levels to 85.7%, while 250 μg/mL and 500 μg/mL led to a marked decrease to 42.8% and 21.5%, respectively. Similarly, in BALB 3T3 cells, *Epavin* at 125 μg/mL reduced ROS levels to 75.6%, while 250 μg/mL and 500 μg/mL resulted in a more significant reduction to 40.9% and 21.3%, respectively. Notably, the reduction in ROS levels was more pronounced in BALB 3T3 cells at lower concentrations, indicating potential differences in sensitivity to oxidative stress and antioxidant defenses between the two models. This observation may be attributed to the inherently higher basal oxidative metabolism and ROS production in hepatocytes, which could require greater antioxidant input to achieve a measurable reduction in oxidative stress.

**Figure 3:**
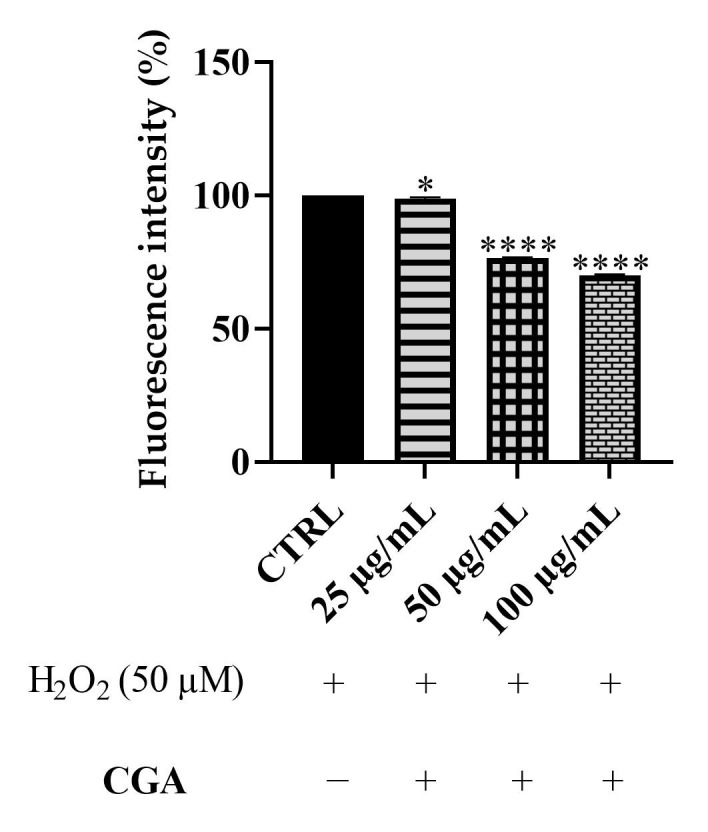
DCFH oxidation in HepG2 cells after exposure to H_2_O_2_, 50 μM, and different concentrations of CGA (25–100 µg/mL). “+” indicates the presence and “−” the absence of treatment. The results are shown as mean ± standard deviation (SD) (*n* = 3). Significant differences versus CTRL: * *p* < 0.05, and **** *p* < 0.0001.

As previously reported for *Epavin*, the eventual cytotoxicity of CGA towards HepG2 cells has been assessed by the mean of the MTT assay. The results indicated that *Epavin* did not affect cell viability till the concentration of 100 μg/mL. These findings are in line with previous studies reporting the cytotoxicity of CGA at concentrations higher than 100 μg/mL [31,32]. Given the promising antioxidant effects observed with *Epavin*, a further investigation was conducted to elucidate the contribution of its principal phytochemical constituent, chlorogenic acid (CGA), as evidenced in the chemical characterization. HepG2 cells exposed to oxidative stress, induced by 50 μM H_2_O_2_, were treated with CGA at concentrations of 25, 50 and 100 μg/mL. As shown in Figure 3, CGA treatment resulted in a concentration-dependent decrease in intracellular ROS levels. Specifically, administration of 25 μg/mL CGA led to a modest yet statistically significant reduction compared to the H_2_O_2_-treated control, whereas 50 and 100 μg/mL treatments produced enhanced decrease in ROS levels, with fluorescence intensities dropping to almost 70% of the control values at the highest concentration. These data indicate that CGA exerts a protective effect against ROS accumulation in HepG2 cells under oxidative stress conditions, although not as pronounced as in *Epavin*. While these results demonstrate a substantial contribution of CGA to the antioxidant activity of *Epavin*, it is important to acknowledge that the overall effect is likely the result of a complex interplay among multiple bioactive constituents present in the formulation. Therefore, although CGA appears to play a pivotal role, the possibility of interactions among different phytochemicals cannot be excluded.

### 3.3. Anti-Inflammatory Activity of Epavin Against LPS-Induced Nitric Oxide Production

**Figure 4:**
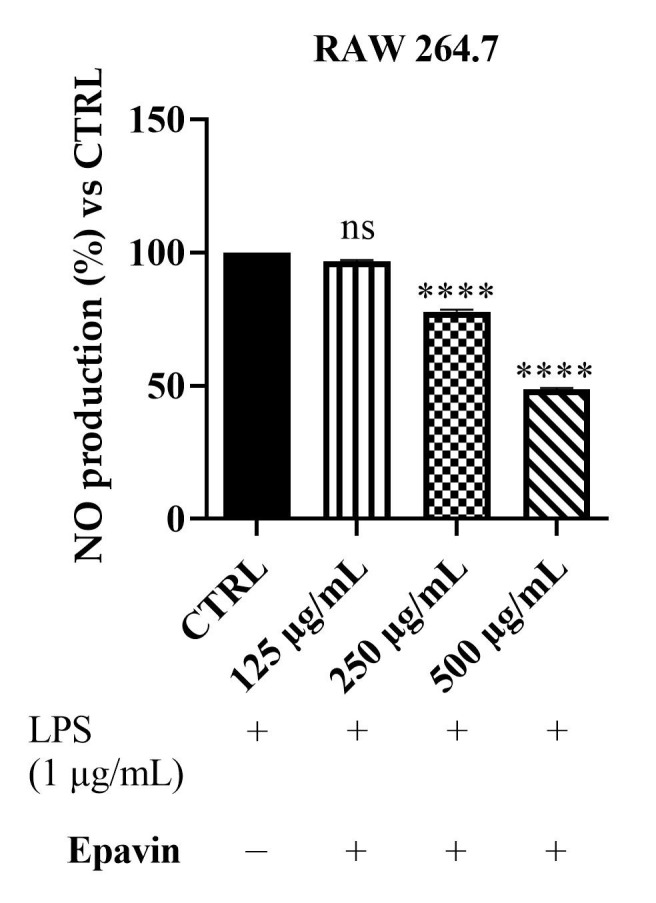
Effect of *Epavin* on nitric oxide (NO) production in LPS-stimulated RAW 264.7 macrophages. The cells were treated with LPS (1 µg/mL) in the presence or absence of *Epavin* at different concentrations (125, 250 and 500 µg/mL). “+” indicates the presence and “−” the absence of treatment. NO levels were measured using the Griess assay and expressed as a percentage relative to the LPS-treated control (the CTRL results are shown as mean ± standard deviation (SD) (*n* = 3). Significant differences versus CTRL: non-significant differences (ns, *p* > 0.05), and **** *p* < 0.0001.

To assess the potential anti-inflammatory effects of *Epavin*, we measured NO production in LPS-stimulated RAW 264.7 macrophages using the Griess assay. As shown in Figure 4, treatment with *Epavin* at 125 µg/mL resulted in a non-significant reduction in NO production (96.7%), whereas at 250 µg/mL and 500 µg/mL, *Epavin* significantly decreased NO levels to 77% and 48%, respectively. These findings indicate that *Epavin* exerts a dose-dependent inhibitory effect on NO production, suggesting a strong anti-inflammatory potential in LPS-induced macrophage activation.

**Figure 5:**
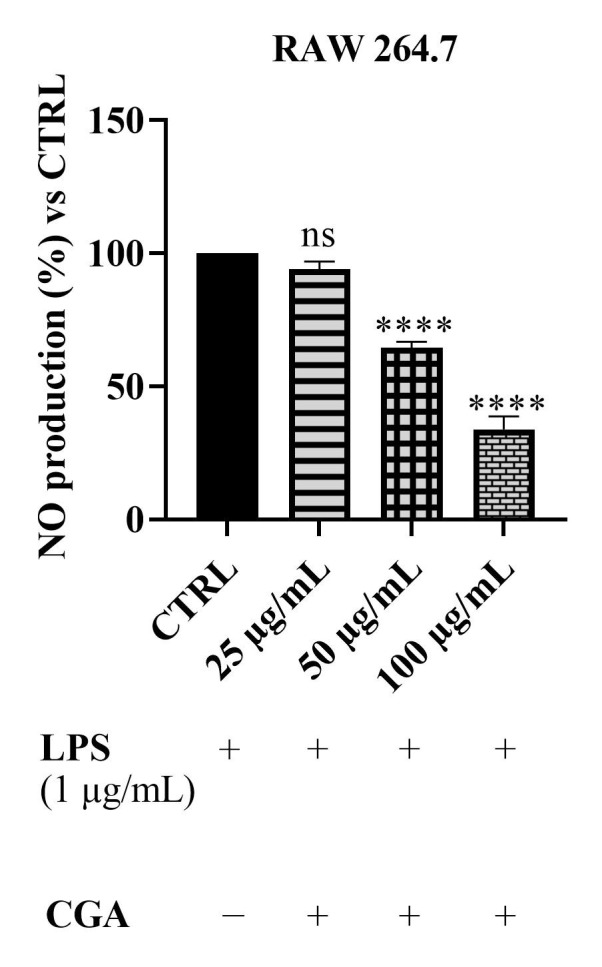
Effect of CGA on nitric oxide (NO) production in LPS-stimulated RAW 264.7 macrophages. The cells were treated with LPS (1 µg/mL) in the presence or absence of CGA at different concentrations (25, 50 and 100 µg/mL). “+” indicates the presence and “−” the absence of treatment. NO levels were measured using the Griess assay and expressed as a percentage relative to the LPS-treated control (CTRL Results are shown as mean ± standard deviation (SD) (*n* = 3). Significant differences versus CTRL: non-significant differences (ns, *p* > 0.05), and **** *p* < 0.0001.

Again, given these results, the effects of CGA were evaluated to determine its specific contribution to the observed anti-inflammatory activity. As depicted in Figure 5, treatment of LPS-stimulated RAW 264.7 cells with CGA at 25, 50 and 100 μg/mL demonstrated a concentration-dependent reduction in NO production. Notably, while 25 μg/mL CGA did not produce a statistically significant effect compared to the LPS control, concentrations of 50 and 100 μg/mL significantly reduced NO levels, with the highest concentration achieving a particularly pronounced inhibition, lowering NO production to approximately 40% of the control value. These data suggest that CGA contributes substantially to the anti-inflammatory properties of *Epavin*, particularly at higher concentrations.

### 3.4. Effects of Cd^2+^, Hg^2+^, and Pb^2+^ on Cell Viability

**Figure 6:**
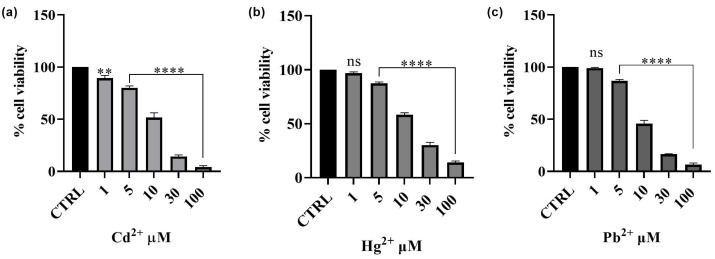
Effects on HepG2 of Cd^2+^ (**a**), Hg^2+^ (**b**), and Pb^2+^ (**c**) (1–100 μM) cell viability after 24 h of exposure. The results are shown as mean ± standard deviation (SD) (*n* = 3). Significant differences versus the control (CTRL): non-significant differences (ns, *p* > 0.05), ** *p* < 0.01, and **** *p* < 0.0001.

Preliminary cytotoxicity assays were conducted to assess the impact of Cd^2+^, Hg^2+^ and Pb^2+^ on human HepG2 hepatoma cells. The results obtained are depicted in Figure 6. The effect of each of the three metals on cell viability was evaluated using the MTT assay. After 24 h of exposure to different concentrations (1–100 µM) of Cd^2+^, Hg^2+^ and Pb^2+^, a dose-dependent cytotoxic effect was observed in the hepatic model. Among the three metals tested, Cd^2+^ exhibited the highest toxicity, followed by Pb^2+^, whereas Hg^2+^ was the least toxic. After treatment with Cd^2+^, the cell survival rate decreased from 89%, at the lowest concentration of 1 μM, to 4%, at the concentration of 100 μM. Pb^2+^ toxicity was observed to be intermediate, with cell viability decreasing from 99 to 7% at the lowest and the highest concentrations tested, respectively. Hg^2+^ did not significantly affect cell viability at lower concentrations (1 μM); however, at the highest concentration tested (100 μM), cell viability decreased to 14%. The findings obtained with this first set of experiments enabled us to select the concentration of metals that notably reduced viability for testing the potential protective effect of *Epavin*. Consequently, the concentration of 30 μM was selected for subsequent experiments, as it induced significant cytotoxicity without completely affecting the cell population. This concentration was chosen for all the metals to ensure that their toxic effects were measurable while still maintaining a sufficient number of viable cells for evaluating potential protective effects. Specifically, at 30 μM, all the metals led to a substantial reduction in cell viability (approximately 14%, 30% and 16% for Cd^2+^, Hg^2+^ and Pb^2+^, respectively), thus allowing for a reliable assessment of both toxicity and potential cytoprotection in later experiments.

### 3.5. Effect of Epavin on Cd^2+^-, Hg^2+^-, and Pb^2+^-Induced Cytotoxicity and ROS Production

**Figure 7:**
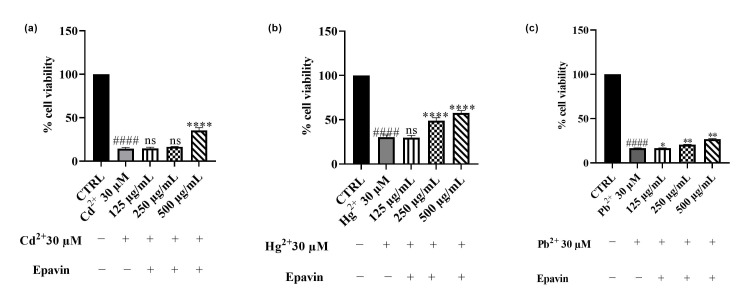
Effects on HepG2 cell viability of Cd^2+^ (**a**), Hg^2+^ (**b**), and Pb^2+^ (**c**), at the concentration of 30 μM; and the effects of co-treatment of Cd^2+^ 30 μM (**a**), Hg^2+^ 30 μM (**b**), and Pb^2+^ 30 μM (**c**), on HegG2 cell viability with different concentration of *Epavin* (125–500 μg/mL) after 24 h of co-treatment. “+” indicates the presence and “−” the absence of treatment. The results are shown as mean ± standard deviation (SD) (*n* = 3). Significant differences in co-treatments versus Cd^2+^, Hg^2+^ or Pb^2+^ alone, respectively: nonsignificant differences (ns, *p* > 0.05), * *p* < 0.05, ** *p* < 0.01, and **** *p* < 0.0001. Significant differences versus control (CTRL): #### *p* < 0.0001.

The potential cytoprotective effects of *Epavin* against HepG2 cells damage induced by Cd^2+^, Hg^2+^ and Pb^2+^ (30 μM) exposure has been assessed. As shown in Figure 7, treatment with 30 μM of heavy metals significantly reduced cell viability compared to the control group (untreated cell, 100% of viability), indicating marked cytotoxicity. Co-treatment with *Epavin* partially counteracted Cd^2+^-induced cytotoxicity in a concentration-dependent manner. Cell viability increased from approximately 13% (Cd^2+^-only treated cells) to nearly 39% at the highest *Epavin* concentration tested. In the case of Pb^2+^ exposure, co-treatment with *Epavin* led to a moderate improvement in cell viability, increasing from around 17% (Pb^2+^-only treated cells) to about 26% at the highest *Epavin* concentration used in the co-treatment. For Hg^2+^ exposure, *Epavin*, the less toxic one of the three heavy metals, exhibited pronounced protective effect. Cell viability rose from about 28% (Hg^2+^-only treated cells) to approximately 58% at 500 μg/mL, indicating a substantial cytoprotective effect.

**Figure 8:**
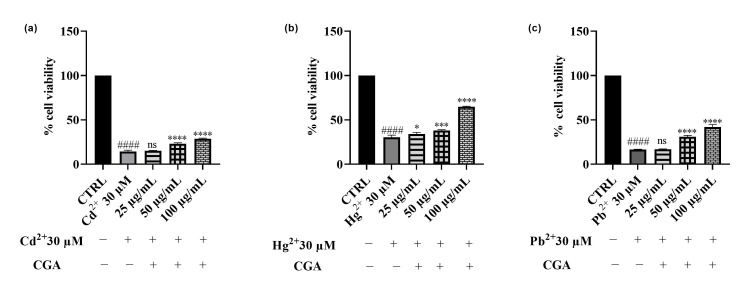
Effects on HepG2 cell viability of Cd^2+^ (**a**), Hg^2+^ (**b**), and Pb^2+^ (**c**), at the concentration of 30 μM, and CGA (25−100 μg/mL) after 24 h of co-treatment. “+” indicates the presence and “−” the absence of treatment. The results are shown as mean ± standard deviation (SD) (*n* = 3). Significant differences versus Cd^2+^, Hg^2+^ or Pb^2+^: nonsignificant differences (ns, *p* > 0.05), * *p* < 0.05, *** *p* < 0.001 and **** *p* < 0.0001. Significant differences versus control (CTRL): #### *p* < 0.0001.

Regarding CGA, hepatic cells exposure to increasing concentrations (25, 50 and 100 μg/mL) of this phytochemical demonstrated a protective effect towards cells treated with all the metals involved, with efficacy varying depending on the metal considered. In the Cd^2+^-treated group (Figure 8a), CGA at 25 μg/mL did not produce a significant protective effect, while 50 and 100 μg/mL significantly improved cell viability, restoring it to approximately 37% and 51%, respectively. These results are consistent with those obtained with the whole *Epavin* extract, suggesting that CGA substantially contributes to the observed protective activity against cadmium-induced toxicity. Similarly, under Pb^2+^ exposure (Figure 8c), CGA led to a concentration-dependent improvement in cell viability, which increased from 18% in Pb^2+^-only treated cells to 42% at 100 μg/mL CGA, indicating a cytoprotective action. Interestingly, in this model as well, the protective profile of CGA closely mirrored that of *Epavin*, albeit with a slightly enhanced efficacy at higher concentrations. In the case of Hg^2+^ exposure (Figure 8b), which resulted in moderate toxicity (approx. 28% viability in the metal-only group), CGA again exerted a significant protective effect. Treatment with 25 μg/mL led to a mild increase in viability (to 44%), whereas 50 and 100 μg/mL substantially restored cell viability to approximately 52% and 78%, respectively. This effect, particularly observed at the highest concentrations, should be highlighted but must certainly be considered in relation to the lower toxicity of mercury.

**Figure 9:**
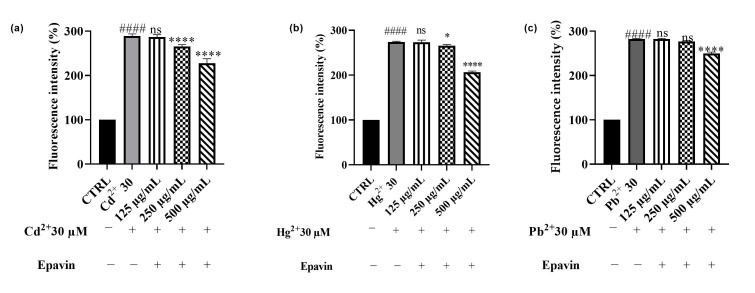
DCFH oxidation in HepG2 cells after exposure to Cd^2+^ (**a**), Pb^2+^ (**b**), or Hg^2+^ (**c**) (30 μM), and different concentrations of *Epavin* (125−500 µg/mL). “+” indicates the presence and “−” the absence of treatment. The results are shown as mean ± standard deviation (SD) (*n* = 3). Significant differences versus Cd^2+^, Hg^2+^ or Pb^2+^: non-significant differences (ns, *p* > 0.05), * *p* < 0.05, and **** *p* < 0.0001. Significant differences versus control (CTRL): #### *p* < 0.0001.

Since heavy metals such Cd^2+^, Hg^2+^ and Pb^2+^ are well-documented inducers of oxidative stress, which plays a central role in their cytotoxic mechanisms, we further investigated whether *Epavin* (Figure 8) and its main phenolic component, CGA (Figure 9), could counteract ROS generation in HepG2 cells under metal-induced stress conditions. The DCFH-DA assay was used to quantify intracellular ROS levels following 12 h co-treatment with 30 µM of each metal and increasing concentrations of either *Epavin* (125–500 µg/mL) or CGA (25–100 µg/mL). In line with their known pro-oxidant effects, all three metals triggered a substantial increase in ROS compared to control cells. Co-treatment with *Epavin* and heavy metals resulted in an observable antioxidant response, which became more appreciable at the highest tested concentration. This effect was particularly evident in cells exposed to Hg^2+^, followed by Cd^2+^, while only a modest reduction in ROS levels was observed in Pb^2+^-treated cells, at the highest concentration.

**Figure 10:**
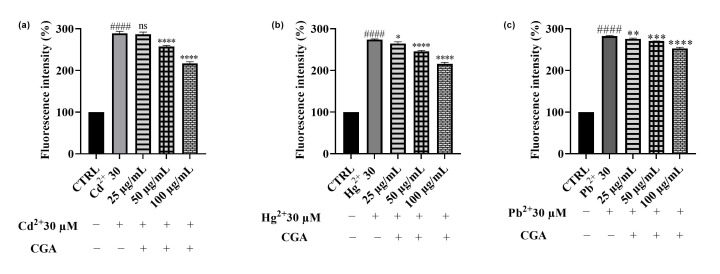
DCFH oxidation in HepG2 cells after exposure to Cd^2+^ (**a**), Pb^2+^ (**b**), or Hg^2+^ (**c**), (30 μM) and different concentrations of CGA (25−100 µg/mL). “+” indicates the presence and “−” the absence of treatment. The results are shown as mean ± standard deviation (SD) (n = 3). Significant differences versus Cd^2+^, Hg^2+^ or Pb^2+^: non-significant differences (ns, *p* > 0.05), * *p* < 0.05, ** *p* < 0.01, *** *p* < 0.001, and **** *p* < 0.0001. Significant differences versus control (CTRL): #### *p* < 0.0001.

A similar trend of results was obtained with CGA (Figure 10) in co-treatment with all the three heavy metals, with the most evident reduction observed at 100 µg/mL, suggesting that CGA significantly contributes to the antioxidant profile of *Epavin*. However, the protective effects of CGA alone were generally comparable but slightly less pronounced than those observed for the full extract, indicating that other constituents within *Epavin* may contribute to its overall efficacy against heavy metal-induced oxidative stress.

### 3.6. Antibacterial Activity of Epavin

The antibacterial activity of *Epavin*, its predominant phenolic compound chlorogenic acid (CGA), and levofloxacin as the reference antibiotic was evaluated according to the Clinical and Laboratory Standards Institute (CLSI) guidelines (document M07-A9, 2012) [12], in order to determine the Minimum Inhibitory Concentration (MIC) and Minimum Bactericidal Concentration (MBC) values. The bacterial panel included a range of clinically relevant Gram-positive and Gram-negative strains, comprising those from the American Type Culture Collection (ATCC) and clinical isolates. The selection of these strains was based on their clinical significance in the context of liver disease and spontaneous bacterial peritonitis, conditions frequently associated with compromised immune responses and increased susceptibility to opportunistic and multidrug-resistant pathogens. The results, expressed as MIC mg/mL, are shown in Table 2. Concerning Gram-positive bacteria, *Epavin,* as a food supplement from natural sources, exhibited potent antibacterial activity, particularly against *Staphylococcus aureus BS*, obtained from clinical isolation, displaying a MIC value of 0.4 mg/mL, and *Staphylococcus aureus* ATCC 25923 with a MIC of 0.7 mg/mL. On the other hand, the methicillin-resistant *S. aureus* (MRSA) ATCC 43300, required a higher concentration (MIC value amounting to 3.0 mg/mL) suggesting a reduced susceptibility. *Enterococcus faecalis* strains showed also a notably susceptibility to *Epavin* action with MIC values of 1.5 mg/mL. Although to lesser extent, it is noteworthy that *Epavin* still exhibited antibacterial activity against the Gram-negative strains used in this study. Indeed, the MIC values for *Escherichia coli* (ATCC 25922 and ESBL-producing BS strain) amounted to 3.0 mg/mL. Similarly, *Klebsiella pneumoniae* strains (ATCC 13883, ATCC 700603, and BS) required higher concentrations, with a MIC value of 6.0 mg/mL indicating a lower susceptibility compared to Gram-positive bacteria. It is noteworthy that for several strains, the MBC values of *Epavin* were equal to the MICs, as observed for *E. faecalis* (ATCC 29212 and BS), *S. aureus* ATCC 25923, and ATCC 29213, while in other cases—such as *S. aureus* ATCC 43300 and most Gram-negative strains—the MBCs differed by only one dilution step from the MICs. This pattern supports the bactericidal action of natural formulation. Concerning CGA, it exhibited moderate antibacterial effects, lower than *Epavin*, with MIC values ranging from 1.5 to 12.0 mg/mL and MBCs from 3.0 to 24.0 mg/mL. Against Gram-positive bacteria, CGA showed its strongest activity against *Staphylococcus aureus* ATCC 25923 (MIC amounting to 1.5 mg/mL), while slightly higher MICs (3.0 mg/mL) were observed for *Enterococcus faecalis* strains and other *S. aureus* isolates. Against Gram-negative strains, although the activity was generally weaker than *Epavin*, except for *Klebsiella pneumoniae* ATCC 13883 and *Klebsiella pneumoniae* ATCC 700603, with MICs of 6.0–12.0 mg/mL, including for resistant isolates such as ESBL-producing *E. coli* and *K. pneumoniae* clinical strains. When compared to CGA, the whole *Epavin* extract consistently exhibited superior antibacterial potency across most tested strains. For example, in *E. faecalis* and *S. aureus* isolates, *Epavin* MICs were twofold lower than those of CGA, and MBC values also reflected enhanced bactericidal capacity. Particularly noteworthy was the *Epavin* activity against clinical *S. aureus* BS, significantly outperforming CGA. Similarly, for Gram-negative strains, although both compounds showed limited activity, *Epavin* consistently presented equal or better MIC/MBC values than CGA.

## 4. Discussion

In recent years, research on hepatoprotective strategies has increasingly shifted toward identifying natural, low-toxicity alternatives capable of complementing or enhancing conventional therapies [33]. While the liver susceptibility to oxidative and inflammatory damage is well recognized, especially in the context of chronic hepatic diseases or exposure to toxic agents, current therapeutic options remain limited, often addressing symptoms rather than the underlying cellular dysfunction.

Natural products, particularly those formulated as dietary supplements, have emerged as promising candidates due to their rich phytochemical profiles and ability to modulate multiple biological pathways simultaneously [34]. Among these, complex botanical blends such as *Epavin*, composed of standardized extracts from plants with traditional and documented hepatotropic activity, could offer a holistic approach to liver support. Indeed, some polyherbal supplements have been explored in recent years for their hepatoprotective potential, reflecting growing interest in plant-based interventions for liver health. Notably, formulations such as AKSS16-LIV01 have demonstrated antioxidant and immunomodulatory effects in animal models of ethanol-induced liver damage, supporting the therapeutic relevance of botanical combinations in hepatic dysfunction [20]. Similarly, Singla et al. evaluated a fermented *Silybum marianum* seed formulation, highlighting its ability to mitigate heavy metal-induced liver injury in rats and reinforcing the importance of phytochemical composition and extraction method on biological efficacy [22].

The results obtained from this study provide insights into the biological activities of *Epavin* in cellular models relevant to hepatic health. By evaluating its effects across different experimental conditions that mimic pathological features commonly associated with CLDs—such as oxidative stress, inflammation, and bacterial insult—we aimed to better understand the multifaceted protective potential of this formulation. The in vitro results obtained from hepatocarcinoma cells (HepG2), macrophages (RAW 264.7), and microbial strains offer a comprehensive perspective on the possible mode of action through which *Epavin* may contribute to liver support and defense. In particular, special attention was given to the role of chlorogenic acid (CGA), a major phenolic constituent, whose individual activity was also assessed to elucidate its contribution to the overall effect. While CGA demonstrated significant antioxidant, anti-inflammatory, and cytoprotective properties, it is important to note that it represents only a fraction of the total phenolic content of the formulation. Consequently, its contribution—though relevant—is unlikely to fully account for the effects observed with the complete extract. Other phytochemicals present in Epavin, such as silymarin flavonolignans (e.g., silybin, silychristin) and dicaffeoylquinic acids, are also known to possess potent hepatoprotective and antioxidant activities [9]. Thus, the observed efficacy of the full formulation likely arises from the combined actions of multiple bioactive constituents, which may act through complementary mechanisms. However, despite the results obtained in this study, some limitations inherent to the in vitro nature of the experimental models must be acknowledged. Indeed, the HepG2 cell line, while commonly employed for hepatotoxicity and oxidative stress research, lacks full metabolic competence and does not accurately mimic the enzyme activity profile of primary human hepatocytes. This may affect both the biotransformation and the intracellular effects of polyphenolic compounds such as those present in Epavin. To overcome these limitations, further investigations using physiologically relevant models are necessary.

### 4.1. ROS Scavenging Effects of Epavin Against H_2_O_2_-Induced Oxidative Stress

The results obtained from the DCFH-DA assay in both HepG2 and BALB 3T3 cells clearly demonstrate the potent ROS-scavenging capacity of *Epavin* in a dose-dependent manner. These findings are consistent with previous reports highlighting the antioxidant potential of multi-herbal formulations, particularly those containing extracts from *Silybum marianum*, *Cynara scolymus*, and *Taraxacum officinale,* all known for their high polyphenolic content and radical-scavenging properties [35]. Notably, our data showed that *Epavin* reduced intracellular ROS levels to nearly 20% with respect to the H_2_O_2_-treated control at the highest concentration tested (500 μg/mL) in both cellular models, suggesting a strong and generalizable antioxidant response. The evaluation of chlorogenic acid (CGA), one of the main bioactive components of *Epavin,* as highlighted by chemical analysis, supports its contribution to the extract overall antioxidant activity. The observed dose-dependent decrease in ROS levels following CGA treatment in HepG2 cells aligns with numerous studies reporting its capacity to modulate redox homeostasis by scavenging free radicals, enhancing endogenous antioxidant enzymes such as superoxide dismutase (SOD), catalase (CAT), and glutathione peroxidase (GPx), and downregulating oxidative-stress-related pathways, including those mediated by NF-κB and MAPK [36]. However, the reduction in ROS levels achieved with CGA itself, although significant, did not fully match the effect of the complete *Epavin* formulation, suggesting the importance of the action of phytocomplex, as is often observed with this type of multi-component natural products. This hypothesis is in line with previous investigations on polyherbal preparations, where the combined activity of different phenolic acids, flavonoids, and terpenoids has been shown to exceed the effects of single compounds, likely due to complementary modes of action, improved bioavailability, or cooperative targeting of multiple molecular pathways [37]. Polyphenols such as silybin and cynarin may exert cytoprotective effects by stabilizing cell membranes and preventing lipid peroxidation, while other alkaloids could contribute to mitochondrial protection and inhibition of ROS-generating enzymes such as NADPH oxidase [38,39]. Additionally, silybin from *Silybum marianum* and cynaropicrin from *Cynara scolymus* have been reported as modulators of the Nrf2 signaling pathway, which regulates the expression of antioxidant response element (ARE)-driven genes [40].

### 4.2. Anti-Inflammatory Activity of Epavin Against LPS-Induced Nitric Oxide Production

The observed dose-dependent inhibition of nitric oxide (NO) production by *Epavin* in LPS-stimulated RAW 264.7 macrophages highlights its promising anti-inflammatory potential. Indeed, macrophages play a crucial role in the immune response, releasing nitric oxide (NO) and various pro-inflammatory cytokines, such as tumor necrosis factor-alpha (TNF-α) and interleukins, in response to inflammatory stimuli [41]. NO, in particular, acts as a key signaling molecule and is synthesized by nitric oxide synthase (NOS) enzymes, including inducible NOS (iNOS), which is upregulated during inflammation. Excessive NO production contributes to oxidative stress and tissue damage, making its regulation essential in controlling inflammation [41]. At concentrations of 250 and 500 µg/mL, *Epavin* significantly reduced NO levels, indicating its ability to modulate inflammatory responses at the cellular level. This finding aligns with the literature reporting the anti-inflammatory effects of various plant-based extracts included in *Epavin*. For instance, *Silybum marianum* (milk thistle) and its major component silybin (A and B) have been shown to inhibit iNOS expression and reduce NO and pro-inflammatory cytokine production in activated macrophages [42]. Similarly, extracts from *Cynara scolymus* (artichoke) have demonstrated anti-inflammatory activity through suppression of NF-κB activation, a key transcription factor involved in the upregulation of iNOS and COX-2 [43]. CGA also exhibited a concentration-dependent inhibition of NO production, confirming its contributory role to the overall anti-inflammatory effect. The potent reduction observed at 100 µg/mL is in agreement with previous studies reporting that CGA suppresses the expression of iNOS and pro-inflammatory cytokines via downregulation of NF-κB and MAPK signaling pathways [44].

### 4.3. Effects of Cd^2+^, Hg^2+^, Pb^2+^, and Epavin on Cell Viability and the Cytoprotective Role of Epavin Against Heavy Metal-Induced Cytotoxicity and ROS Production

The results of this study provide compelling evidence for the cytoprotective and antioxidant properties of *Epavin* against heavy metal-induced toxicity in HepG2 cells. Exposure to Cd^2+^, Pb^2+^, and Hg^2+^, known environmental pollutants with hepatotoxic potential, led to a significant decline in cell viability and a marked increase in ROS levels, confirming their ability to induce oxidative stress-mediated damage. Notably, co-treatment with *Epavin* significantly mitigated these detrimental effects in a concentration-dependent manner, with the most prominent protective effects observed against Hg^2+^ and Cd^2+^ toxicity. These findings are consistent with previous studies demonstrating the hepatoprotective potential of polyherbal formulations rich in polyphenols and flavonoids, which are capable of scavenging ROS and enhancing endogenous antioxidant defenses [45]. The results on CGA, one of the major components in the phytocomplexes, highlighted its protective role in this context. Particularly at higher concentrations, CGA exhibited substantial cytoprotective and antioxidant effects, especially under Hg^2+^-induced stress, where it restored cell viability by nearly 50% compared to the metal-only treated cells. Our results support previous studies describing CGA capacity to chelate metal ions, reduce lipid peroxidation, and modulate intracellular redox balance [44,46,47]. Moreover, CGA has been shown to alleviate Cd^2+^-induced hepatorenal oxidative injury in mice by reducing NO and myeloperoxidase production [48]. Similarly, it has demonstrated neuroprotective effects against Cd^2+^-induced oxidative neuropathy in murine models by limiting lipid peroxidation, restoring glutathione levels, and stabilizing mitochondrial membranes [49]. However, while CGA itself was effective, the slightly superior efficacy observed with the complete *Epavin* formulation suggests a potential interplay among its multiple bioactive components.

### 4.4. Antibacterial Activity of Epavin

The antibacterial activity observed for *Epavin* and its principal component, chlorogenic acid (CGA), aligns with the existing literature, underscoring the potential of natural phenolic compounds in counteracting bacterial pathogens. In our study, *Epavin* exhibited notable efficacy against Gram-positive bacteria, particularly *Staphylococcus aureus*, with MIC values as low as 0.4 mg/mL for clinical isolates. This potency was higher than that of the main component CGA, which demonstrated MICs ranging from 1.5 to 12.0 mg/mL, indicating that, also in this case, the overall antibacterial effect of *Epavin* likely results from the combined action of its various bioactive constituents. Comparable studies have reported similar MIC values for CGA against *S. aureus*. For instance, CGA showed a MIC of 2.5 mg/mL against *S. aureus* [50], and other work found MICs ranging from 0.6 to 10.0 mg/mL for several Gram-positive and Gram-negative strains, including *K. pneumoniae* [51]. Moreover, previous investigations reported that ethanol and methanol extracts of *Silybum marianum* exhibited MIC values against *Staphylococcus aureus* in the range of 0.125–0.5 mg/mL (e.g., 0.5 mg/mL for free silymarin, reduced to 0.125 mg/mL when nano-encapsulated [52], while ethanolic extracts of *Cynara scolymus* demonstrated MICs of approximately 3.1 mg/mL [53]. These findings confirm that Epavin antibacterial efficacy—particularly the MIC of 0.4 mg/mL against clinical *S. aureus*—is consistent with, and in some cases exceeds, the activity of its main botanical constituents. Although it is not possible to attribute the overall activity of *Epavin* solely to its main constituent, the results obtained with CGA itself clearly highlight the contribution of this bioactive compound to the total observed effect. The activity of this food supplement may be attributed to the presence of other phenolics and secondary metabolites that contribute to antibacterial action through multiple mechanisms. In the case of Gram-negative bacteria, *Epavin* showed moderate activity, with MIC values of 3.0 mg/mL for *Escherichia coli* and 6.0 mg/mL for *Klebsiella pneumoniae*. While this MIC value seems to be above the typical thresholds of clinical relevance, it is important to emphasize that strains as *K. pneumoniae* are intrinsically difficult to target due to their low-permeability outer membrane, active efflux systems, and frequent multidrug resistance, which make them particularly challenging to treat. In light of this, it is not trivial to observe antibacterial activity against this species using natural product-based formulations. Hence, finding natural compounds with activity against these pathogens remains a valuable and encouraging result. The MBC values were generally close to the MICs (within one dilution), indicating that *Epavin* exerts bactericidal rather than merely bacteriostatic effects. Mechanistically, CGA has been reported to compromise membrane integrity, disturb metabolic enzymes such as succinate and malate dehydrogenase, and even reduce bacterial virulence and biofilm formation [54]. These mechanisms, together with those potentially exerted by other phenolics in *Epavin*, may explain its greater antibacterial efficacy. Overall, these findings support the potential of *Epavin* as a source of natural antibacterial agents. The combined effects of its constituents contribute to an enhanced antimicrobial profile compared to CGA alone, particularly against Gram-positive pathogens.

## 5. Conclusions

This study investigated the biological properties of the polyherbal formulation *Epavin*, with a focus on its antioxidant, anti-inflammatory, cytoprotective, and antibacterial activities, using in vitro models relevant to hepatic function. *Epavin* exhibited dose-dependent antioxidant effects in both hepatic (HepG2) and fibroblast (BALB 3T3) cell lines subjected to oxidative stress, with results indicating a substantial contribution of chlorogenic acid (CGA), its major phenolic constituent. Similarly, *Epavin* demonstrated anti-inflammatory activity in LPS-stimulated RAW 264.7 macrophages by reducing NO production, with CGA conceivably playing a supportive but not exclusive role. Under conditions of heavy metal-induced cytotoxicity, *Epavin* conferred measurable protection, particularly against Cd^2+^ and Hg^2+^ toxicity, which was accompanied by a reduction in intracellular ROS levels.

Antibacterial testing revealed that *Epavin* exerted bactericidal effects, especially against Gram-positive strains. These findings suggest a combined contribution of various phytochemicals in the extract to the observed bioactivities, although the specific molecular interactions and mechanisms were not fully elucidated. Herein, the results highlight that *Epavin*, as a food supplement composed of traditionally hepatoprotective botanical extracts, may represent a potential adjuvant agent in therapeutic strategies for liver health management and hepatic dysfunction.

The multifaceted experiments designed in this study allowed us to highlight the multiple biological activities of this food supplement relevant to hepatic health and protection. Although the current work includes only in vitro models, the obtained data represent a valuable starting point for further studies aimed at deepening the pharmacokinetic profile in order to support its biological relevance in vivo.

## Figures and Tables

**Table 1:** Chemical characterization results for *Epavin*.

Peak n.	Compound	Rt (min)	µg/mL ± SD	UV (λ max, nm)	[M-H] (*m*/*z*)	ESI-MS/MS (%)
1	Silychristin	13.48	0.48 ± 0.02	287.8; 325.8	481	451 (5); 301 (18); 285 (24); 283 (8); 273 (14); 257 (7); 169 (21)
2	Silydianin	14.17	0.46 ± 0.02	272.5; 302.1	481	451 (5); 435 (9); 301 (18); 285 (24); 283 (8); 273 (14); 257 (7); 169 (21)
3	Chlorogenic acid	16.62	3.30 ± 0.04	299; 327	353	191 (100)
4	Silybin A	18.43	0.54 ± 0.03	287.8; 322.2	481	451 (5); 435 (9); 301 (18); 285 (24); 273 (14); 257 (7); 169 (21)
5	Silybin B	25.28	0.47 ± 0.06	283.1; 328.2	481	451 (5); 435 (9); 301 (18); 285 (24); 283 (8); 273 (14); 169 (21)
6	Vitexin	25.71	0.67 ± 0.04	268.9; 337.7	431	311 (25); 269 (100)
7	Luteolin-7-*O*-glucuronide	26.04	0.93±0.09	266.6;298.3; 336.5	461	341 (5); 285 (16)
8	Cinaroside	27.31	2.30 ± 0.10	256; 265.4; 347.2	447	327 (1) [M-120-H]^−^; 285 (100) [M-162-H]^−^
9	Cynarine	28.77	2.25 ± 0.11	300; 325.8	515	353 (18); 191 (76); 179 (23); 173 (21); 135 (18)
10	Apigenin-7-*O*-glucoside	29.48	0.79 ± 0.06	281.9; 334.1	431	269 (100)
11	3,4-dicaffeoylquinic acid	30.43	2.68 ± 0.06	300.2; 329.4	515	353 (18); 191 (76); 179 (23); 173 (21); 135 (18)
12	4,5-dicaffeoylquinic acid	31.62	1.03 ± 0.07	300.9; 327	515	353 (18); 191 (76); 179 (23); 173 (21); 135 (18)
13	Luteolin	38.93	0.95 ± 0.13	254; 268; 349.5	285	133 (100)

**Table 2:** Minimum inhibitory concentration (MIC, mg/mL) and minimum bactericidal concentration (MBC, mg/mL) of *Epavin*, CGA, and the reference antibiotic levofloxacin (MIC, µg/mL).

	*Epavin*		CGA		Levofloxacin
Gram-Positive Strains	MIC	MBC	MIC	MBC	MIC
*E. faecalis* ATCC 29212	1.5	1.5	3.0	6.0	2
*E. faecalis* BS	1.5	1.5	3.0	6.0	8
*S. aureus* ATCC 25923	0.7	0.7	1.5	3.0	0.5
*S. aureus* ATCC 29213	1.5	1.5	3.0	6.0	0.5
*S. aureus* ATCC 43300	3.0	6.0	6.0	12.0	1
*S. aureus BS*	0.4	0.8	1.5	3.0	1
**Gram-negative strains**					
*Escherichia coli* ATCC 25922	3.0	6.0	6.0	12.0	0.02
*Escherichia coli* BS (ESBL)	3.0	6.0	6.0	12.0	1
*Klebsiella pneumoniae* ATCC 13883	6.0	12.0	6.0	12.0	8
*Klebsiella pneumoniae* ATCC 700603	6.0	12.0	6.0	12.0	8
*Klebsiella pneumoniae* BS	6.0	12.0	12.0	24.0	32

## Data Availability

The original contributions presented in this study are included in the article. Further inquiries can be directed to the corresponding authors.
